# Correlation of Vitamin D with Inflammatory Cytokines, Atherosclerotic Parameters, and Lifestyle Factors in the Setting of Heart Failure: A 12-Month Follow-Up Study

**DOI:** 10.3390/ijms20225811

**Published:** 2019-11-19

**Authors:** Daniel N. Roffe-Vazquez, Anna S. Huerta-Delgado, Elena C. Castillo, José R. Villarreal-Calderón, Adrian M. Gonzalez-Gil, Cecilio Enriquez, Gerardo Garcia-Rivas, Leticia Elizondo-Montemayor

**Affiliations:** 1 Tecnologico de Monterrey, Center for Research in Clinical Nutrition, Escuela de Medicina, Monterrey 64710, N.L., Mexico; daniel.roffe@hotmail.com (D.N.R.-V.); anna.sofy@hotmail.com (A.S.H.-D.); joser.villarreal@udem.edu (J.R.V.-C.); amgonzalezgil@hotmail.com (A.M.G.-G.); 2 Tecnologico de Monterrey, Centro de Investigacion Biomedica, Hospital Zambrano Hellion, San Pedro Garza-Garcia 66278, N.L., Mexico; ecgonzalez@tec.mx (E.C.C.); dr.cecilioenriquez@medicos.tecsalud.mx (C.E.); 3 Tecnologico de Monterrey, Cardiovascular and Metabolomics Research Group, Escuela de Medicina, San Pedro Garza-Garcia 66278, N.L., Mexico

**Keywords:** vitamin D, heart failure, inflammation, seasonal variation, lifestyle, cytokines, lipids, mechanisms, immunoregulatory

## Abstract

Vitamin D deficiency is highly prevalent worldwide. It has been associated with heart failure (HF) given its immunoregulatory functions. In-vitro and animal models have shown protective roles through mechanisms involving procollagen-1, JNK2, calcineurin/NFAT, NF-κB, MAPK, Th1, Th2, Th17, cytokines, cholesterol-efflux, oxLDL, and GLUT4, among others. A 12-month follow-up in HF patients showed a high prevalence of vitamin D deficiency, with no seasonal variation (64.7–82.4%). A positive correlation between serum 25(OH)D concentration and dietary intake of vitamin D-rich foods was found. A significant inverse correlation with IL-1β (*R* = −0.78), TNF-α (*R* = −0.53), IL-6 (*R* = −0.42), IL-8 (R = −0.41), IL-17A (*R* = −0.31), LDL-cholesterol (*R* = −0.51), Apo-B (*R* = −0.57), total-cholesterol (*R* = –0.48), and triglycerides (*R* = −0.32) was shown. Cluster analysis demonstrated that patients from cluster three, with the lowest 25(OH)D levels, presented the lowermost vitamin D intake, IL-10 (1.0 ± 0.9 pg/mL), and IL-12p70 (0.5 ± 0.4 pg/mL), but the highest TNF-α (9.1 ± 3.5 pg/mL), IL-8 (55.6 ± 117.1 pg/mL), IL-17A (3.5 ± 2.0 pg/mL), total-cholesterol (193.9 ± 61.4 mg/dL), LDL-cholesterol (127.7 ± 58.2 mg/dL), and Apo-B (101.4 ± 33.4 mg/dL) levels, compared with patients from cluster one. Although the role of vitamin D in the pathogenesis of HF in humans is still uncertain, we applied the molecular mechanisms of in-vitro and animal models to explain our findings. Vitamin D deficiency might contribute to inflammation, remodeling, fibrosis, and atherosclerosis in patients with HF.

## 1. Introduction

It has long been recognized that vitamin D plays a major role in bone and mineral metabolism, but until recent years, it has also been implicated in immunity and cardiovascular health. The first rate-limiting step in the endogenous synthesis of vitamin D is the skin’s sun exposure, as ultraviolet B (UVB) radiation is necessary to convert 7-dehydrocholesterol into vitamin D3 (cholecalciferol), with estimates of cutaneous synthesis providing between 80% and 100% of the vitamin D requirements of the body [1]. In the liver, 25-hydroxylase (CYP2R1) converts vitamin D3 into 25-hydroxyvitamin D [25(OH)D], which is then transformed in the kidney by means of 1-alpha-hydroxylase (CYP27B1), into 1,25-dihydroxyvitamin D [1,25(OH)2D3], also known as calcitriol, the hormone’s biologically active form [2]. The enzyme 1-alpha-hydroxylase is activated by the parathyroid hormone (PTH) and inhibited by fibroblast growth factor-23 (FGF-23). On the other hand, 25-hydroxyvitamin D3 24-hydroxylase (CYP24A1) is involved in calcitriol’s catabolism. This enzyme limits the generation of calcitriol by converting it into hydroxylated derivatives (which will later be excreted), or by converting 25(OH)D3 into 24,25(OH)2D3 or 23,25(OH)2D3, further decreasing its availability for 1-hydroxylation. CYP24A1 is inhibited by PTH and induced by FGF-23 [3]. By feedback mechanisms, both 1-alpha-hydroxylase and 25-hydroxyvitamin D3 24-hydroxylase regulate 1,25(OH)2D3′s bioavailability as a means to protect against hypercalcemia [3,4].

Several risk factors have been described to predispose to vitamin D deficiency. Living in higher latitudes (above 35 degrees) is a well-known risk factor [5]; however, vitamin D deficiency has been shown to be highly prevalent even in populations living near the equator [6]. Other risk factors for vitamin D deficiency include skin phototype, sunscreen usage, indoor working environments, minimal outdoor activity, obesity, high body mass index (BMI), and waist circumference (WC), as well as older age, which is accompanied by a 75% decrease in vitamin D synthesis capacity [1,6]. Among these factors, the combination of low UVB availability and/or underexposure, in addition to insufficient dietary intake, have been described as the most important [1].

Notably, nutrition surveillance data from various countries have indicated that vitamin D ingestion is lower than the recommended minimum amount [1,7]. There is also evidence that suggests that the recommended intake established by guidelines (600 IU) is actually insufficient and should be increased to at least 800 IU [5].

Vitamin D also plays a role in immune regulation, as immune cells express 1-alpha-hydroxylase to regulate their own local concentration of calcitriol. This effect has been related to the milieu of cytokines [8], given that adequate levels of serum 25(OH)D have been associated with increased levels of the anti-inflammatory cytokines interleukins (IL) 4 and 10, and to lower levels of the proinflammatory cytokines IL-1 and IL-6 [9]. On the contrary, vitamin D deficiency has been associated with chronic inflammatory states and a proinflammatory cytokine profile. Vitamin D deficiency has also been linked with augmented collagen synthesis, oxidative stress, and fibrosis, which are potential mechanisms underlying cardiovascular diseases, such as heart failure [10].

Heart failure (HF) is one of the most common chronic medical conditions worldwide. It is estimated that as of 2019, more than 6 million Americans are living with this disease [11,12,13]. HF with reduced ejection fraction (HFrEF) is defined by a left ventricular ejection fraction (LVEF) of < 40% [14]. Most of the risk factors for HFrEF in Western populations have been described in association with ischemic heart disease [15]. It has been proposed that vitamin D might play a role in disease pathogenesis, as serum 25(OH)D levels have been demonstrated to be lower in patients with HF compared with control subjects [16]. Specifically, prospective studies have shown that the risk of developing HF is increased in patients with vitamin D deficiency [17]. One of the mechanistic links regarding this association relies on a proinflammatory cytokine state, including both innate and adaptive immunity [18,19,20].

Regarding innate immunity, vitamin D deficiency has been shown to increase the expression of nuclear-factor kappa B (NF-κB), leading to a higher secretion and release of the main proinflammatory cytokines, such as IL-6 and monocyte chemoattractant protein-1 (MCP-1) [10]. Cardiomyocytes themselves are activated by hemodynamic stress and are able to secrete several inflammatory cytokines as well [21]. On the other hand, by activation of mitogen-activated protein kinase (MAPK) phosphatase-1 and subsequent inhibition of p38 MAPK, vitamin D has been demonstrated to switch the cytokine profile into an anti-inflammatory state, with inhibition of the production of tumor necrosis factor-alpha (TNF-α) and IL-6 [22]. In the case of adaptive immunity, vitamin D has been found to inhibit the CD4+ T-cell course towards the Th17 lineage [9]. Additionally, vitamin D is able to hinder IL-17 production of those CD4+ T-cells that have been already committed towards the Th17 lineage [23]. Not only does vitamin D exert its effects through immune regulation, but it is also able to control extracellular matrix metabolism. Vitamin D deficiency has been related to increased expression of matrix metalloproteinase (MMP) 2 and 9 [10], as well as to augmented tissue macrophage infiltration [24]. These mechanisms may be associated with some of the structural abnormalities seen in HF, such as ventricular remodeling, tissue fibrosis, and a systemic proinflammatory state. Other mechanisms through which vitamin D deficiency predisposes to HF include: overactivation of the renin-angiotensin-aldosterone system (RAAS); dysfunction of the intracellular calcium handling by the cardiomyocyte; overexpression of procollagen-1, which leads to increased fibrosis; diminished protein kinase A (PKA) levels that result in impaired contractility; and activation of calcineurin signaling, which promotes cardiac hypertrophy [25,26,27,28].

Given the evidence provided relating the mechanisms through which vitamin D deficiency is associated with HF, it is important to study the seasonal variation of vitamin D deficiency and the cytokine profile, as well as the lifestyle factors that exert an influence in patients with HF. Thus, the aim of this study is to describe the seasonal variation of vitamin D deficiency and its association with 13 inflammatory cytokines, biochemical, and lifestyle factors during a 12-month follow-up in a cohort of patients with HFrEF, as well as to describe the molecular inflammatory and atherosclerotic mechanisms related to vitamin D deficiency. An original figure summarizing these mechanisms is also shown.

## 2. Results

### 2.1. Demographic, Anthropometric, and Lifestyle Parameters

The mean and standard deviation of serum 25(OH)D concentrations, demographic data, lifestyle, and anthropometric characteristics are presented in Table 1. The mean age of the cohort (*n* = 17) was 64.2 years. Most of the patients in the study were males (82.4%). In order to assess the level of skin pigmentation in our patients, the Fitzpatrick’s classification of phototypes was clinically determined. Most of the patients belonged to phototype IV (58.8%), followed by V (29.4%), and lastly by phototype III (11.8%). All of the patients were of Hispanic ethnicity. Heart failure severity was clinically determined by the New York Heart Association (NYHA) classification of functional capacity [29]. All of the patients in our cohort had an NYHA class III status, which describes subjects that are asymptomatic at rest but develop fatigue, dyspnea, or palpitations when performing less than ordinary physical activity. Mean BMI was 28.5 kg/m^2^, body fat percentage (BF%) was 30.5%, WC was 99.1 cm, and fat mass was 23.4 kg.

**Figure 1:**
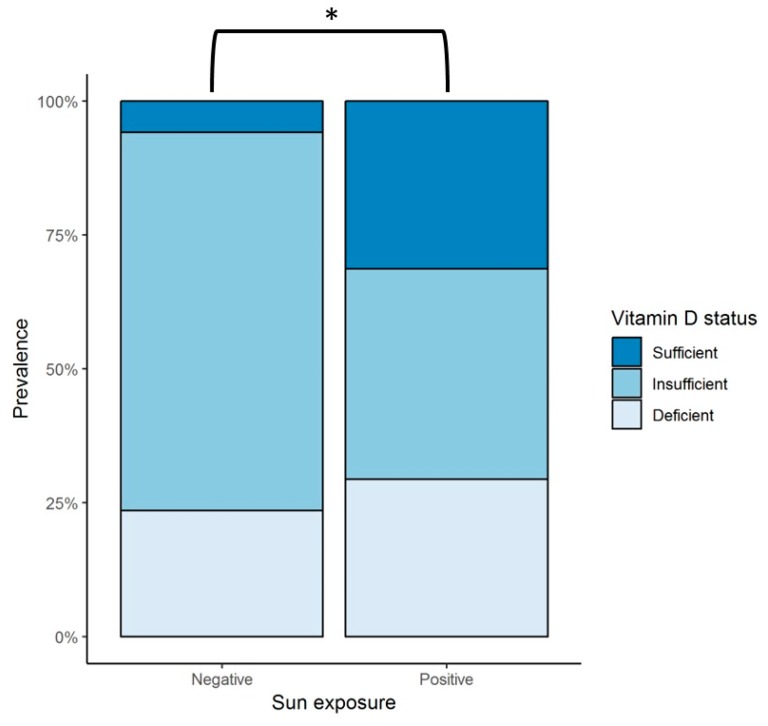
Sun exposure and vitamin D status in patients with heart failure during the 12-month follow up. Negative = observations with null sun exposure; positive = observations with any amount of sun exposure; * represents a statistically significant difference in the prevalence of vitamin D sufficiency in patients with some amount of sun exposure (positive), 31.4%, versus those with null exposure (negative), 5.9% (*p* < 0.044). Sufficient vitamin D status is defined as serum 25(OH)D levels > 30 ng/mL, insufficient as serum 25(OH)D levels > 20 and < 30 ng/mL, and deficient status as serum 25(OH)D levels < 20 ng/mL. [30]. 25(OH)D = 25-hydroxyvitamin D.

Mean vitamin D intake per day was calculated to be 224 ± 113.1 IU; all of the patients were found to have an overall consumption throughout the year of less than 400 IU/day. Sun exposure had a large variability among subjects; therefore, this data was further analyzed by grouping patients into those who reported a null amount of sun exposure (negative) versus those who had any time of sun exposure (positive) per day (Figure 1). Since individual patients reported different amounts of sun exposure throughout the seasons, the data was analyzed considering four observations per patient, each corresponding to a season of the year, with a total of 68 observations. From these, 51 of the observations were categorized as positive and 17 as negative. A vitamin D sufficient status was statistically more prevalent in patients with sun exposure (31.4%) compared to those with no exposure (5.9%) (*p* < 0.044).

### 2.2. Cluster Analysis

**Figure 2:**
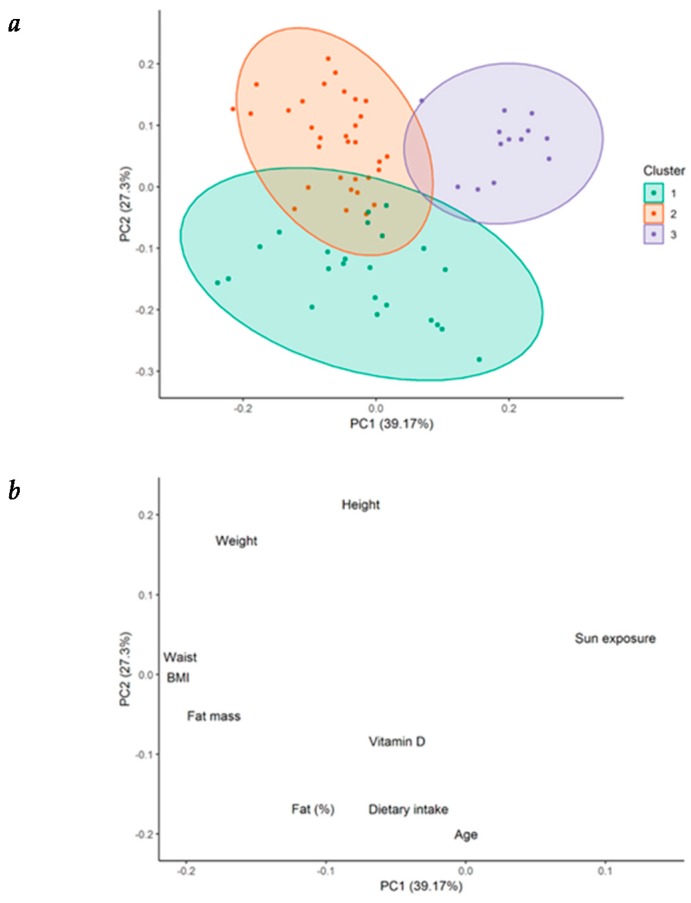
Cluster partitioning in patients with heart failure. (**a**) Cluster partitioning and its graphical representation of the principal component analysis (PCA) in patients with heart failure (HF); (**b**) graphical representation of the contribution of each individual variable to the variance of the PCA. BMI = body mass index.

Principal component analysis (PCA) was performed with numeric demographic, anthropometric, and lifestyle variables, as well as with serum 25(OH)D concentration, in order to better understand the nature of the observations in our cohort. The 68 observations (four observations per patient, each one according to the corresponding season) were classified into three clusters by partitioning around medoids (PAM). Graphical representation of the grouping of each observation is shown in Figure 2a, while the contribution of each variable to the total variance of PCA is seen in Figure 2b.

#### 2.2.1. Cluster Analysis for Lifestyle and Anthropometric Parameters

**Figure A1:**
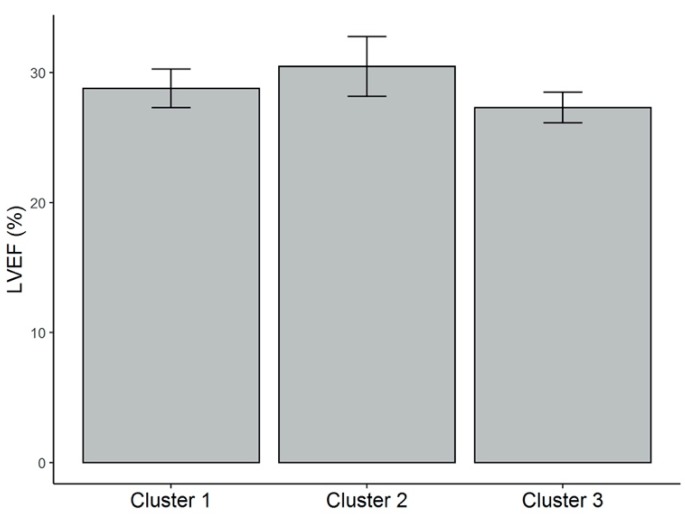
LVEF (%) values for each of the clusters. Mean and standard error (vertical bars) are shown. There was no significant difference of this parameter among clusters. LVEF = left ventricular ejection fraction.

Differences in data of patients grouped into three clusters is summarized in Table 2. Patients from cluster 1 were characterized by the highest levels of serum 25(OH)D concentration (28.3 ± 8.0 ng/mL). These patients presented the highest vitamin D dietary intake (322.9 ± 103.4 IU/day), in spite of being older (72.2 ± 7.5 years), as well as the highest BF% (35.8% ± 4.9%), compared with patients from clusters 3 and 2. However, the total fat mass in kg and the BMI were significantly different from those shown by patients from cluster 3, but not from those of cluster 2. In contrast, patients from cluster 3 presented the lowest serum 25(OH)D levels (19 ± 4.5 ng/mL). These patients showed the highest amount of sun exposure (1178.5 ± 925.6 min/week) compared with patients from the other two clusters, but lower vitamin D ingestion (174 ± 72.9 IU/day) compared with patients from cluster 1. They also displayed lower BMI (24.6 ± 1.2 kg/m^2^), BF% (22.7% ± 3.3%), and fat mass in kg (15.0 ± 2.6 kg) than patients from cluster 1. Finally, patients from cluster 2 showed the highest body weight (85.4 ± 8.0 kg) and WC (103.9 ± 7.1 cm); lower vitamin D intake (173.1 ± 83.9 IU/day), but no difference in sun exposure compared with patients from cluster 1. No significant difference among the clusters was demonstrated for LVEF (%): cluster 1 (28.8% ± 7.1%), cluster 2 (30.5% ± 13.0%), cluster 3 (27.3% ± 4.3%) (Figure A1-Appendix A).

#### 2.2.2. Biochemical and Metabolic Parameters among the Clusters of Patients with HF

**Figure 3:**
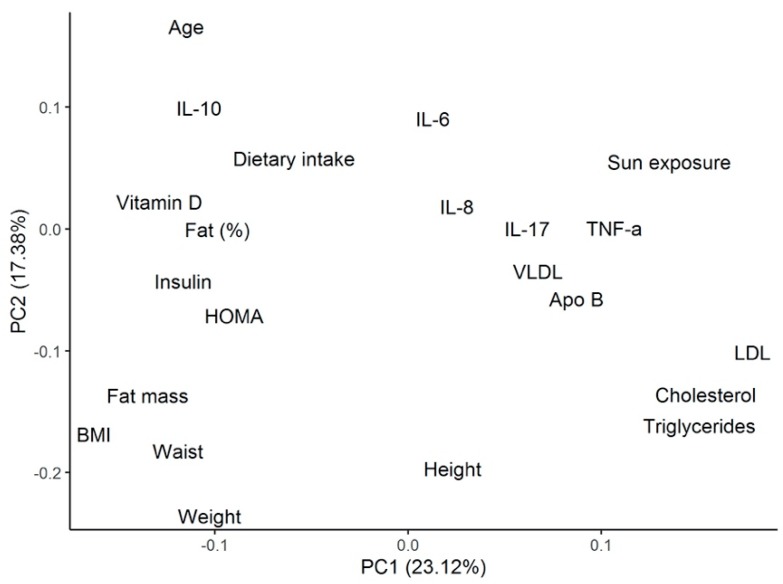
PCA analysis for the metabolic and biochemical variables described in the cluster’s analysis. PCA = principal component analysis; IL= interleukin; HOMA = Homeostatic Model Assessment; BMI = body mass index; TNF = tumor necrosis factor; VLDL = very low-density lipoprotein; Apo = apolipoprotein; LDL = low-density lipoprotein.

Using the same cluster classification, Table 3 shows the differences among clusters for biochemical and metabolic parameters. Patients from cluster 3 were found to present with the highest levels of total cholesterol (TC) (193.9 ± 61.4 mg/dL), low-density lipoprotein (LDL) cholesterol (127.7 ± 58.2 mg/dL), non-high-density lipoprotein (non-HDL) cholesterol (152.3 ± 58.2 mg/dL), Apo-B (101.4 ± 33.4 mg/dL), Apo-B/Apo-A ratio (0.7 ± 0.2), and TC/HDL ratio (4.7 ± 1.4). All of these metabolic parameters were significantly different compared with patients from cluster 1. These patients also presented the lowest insulin levels (8.6 ± 4.5 µU/mL) and Homeostatic Model Assessment (HOMA) index (2.7 ± 1.6), both of which were significantly different regarding cluster 2. Figure 3 shows the PCA, including the biochemical and metabolic variables shown in Table 3.

#### 2.2.3. Cytokine Differences among the Clusters of Patients with Heart Failure

Cytokine profile analysis, according to the patient’s cluster grouping, is shown in Table 4. Patients from cluster 1 showed the lowest levels of TNF-α (3.3 ± 4.1 pg/mL) and IL-17A (2.4 ± 2.3 pg/mL), as well as the highest levels of IL-10 (2.7 ± 2.0 pg/mL), IL-6 (6.2 ± 6.9 pg/mL), IL-18 (471.2 ± 400.6 pg/mL), IL-12p70 (4.5 ± 6.0 pg/mL), and interferon (IFN)-α2 (74.5 ± 99.7 pg/mL). Among these cytokines, TNF-α, IL-12p70, IFN-α2, and IL-10 were significantly different from cluster 3. Opposite to these results, patients from cluster 3 showed the highest levels of TNF-α (9.1 ± 3.5 pg/mL), IL-8 (55.6 ± 117.1 pg/mL), and IL-17A (3.5 ± 2.0 pg/mL), as well as the lowest levels of IL-10 (1.0 ± 0.9 pg/mL), IL-12p70 (0.5 ± 0.4 pg/mL), and IFN-α2 (1.1 ± 0.4 pg/mL). From these, TNF-α, IL-12p70, IL-10, and IFN-α2 levels were significantly different regarding cluster 1. Patients from cluster 2 were shown to have significant lower levels of IL-10 (1.1 ± 0.8 pg/mL), IL-12p70 (0.6 ± 0.6 pg/mL), and IFNα-2 (4.3 ± 6.5 pg/mL) compared with those from cluster 1. Figure 3 shows the PCA, including the cytokine variables shown in Table 4.

### 2.3. Correlation between 25(OH)D Levels and Ventricular Function, Biochemical, Lifestyle, and Anthropometric Parameters in Patients with Heart Failure

**Figure A2:**
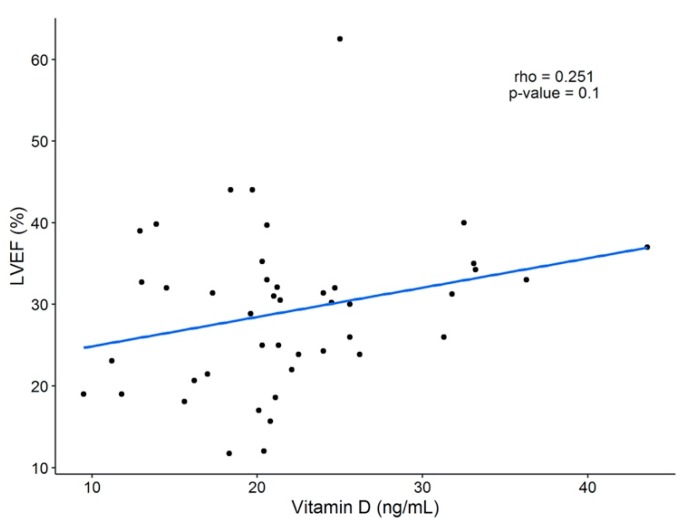
Linear correlation between LVEF (%) and 25(OH)D concentration. A weak positive correlation was found (*R* = 0.251), but no statistical significance was seen. LVEF = left ventricular ejection fraction; 25(OH)D = 25-hydroxivitamin D.

A significant positive correlation was found between 25(OH)D serum levels and age (*R* = 0.386, *p* < 0.01), BMI (*R* = 0.265, *p* < 0.05), dietary intake of vitamin D-rich foods (*R* = 0.276, *p* < 0.05), and calcium (*R* = 0.354, *p* < 0.05) (Table 5 and Table 6). No association was observed between sun exposure and 25(OH)D concentration when the total data was analyzed; however, as was shown in Figure 1, a difference was demonstrated when the observations were subgrouped into null sun exposure or any time of sun exposure. There was no correlation between 25(OH)D levels and ventricular function parameters (LVEF and brain natriuretic peptide [BNP]) (Table 6 and Figure A2-Appendix A).

### 2.4. Lipid Parameters Are Negatively Correlated with 25(OH)D Levels in Patients with Heart Failure

**Figure 4:**
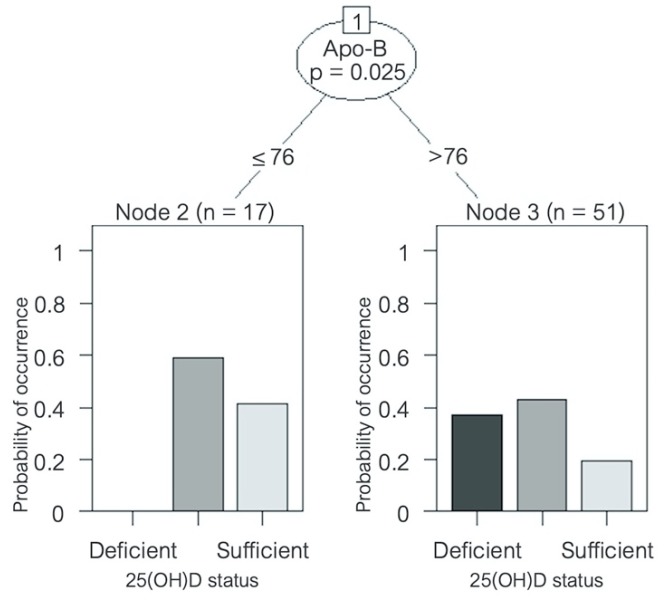
Decision tree predicting vitamin D status in patients with heart failure. Variable name and *p*-value is shown inside the circle; the corresponding lines show cut-off values. Bar graphs at the bottom show 25(OH)D status distribution amongst patients. Apo = apolipoprotein; sufficiency = 25(OH)D ≥ 30 ng/mL, insufficiency = 25(OH)D ≥ 20 and < 30 ng/mL, and deficiency = 25(OH)D < 20 ng/mL [30]. 25(OH)D = 25-hydroxyvitamin D.

A significant inverse correlation was found between serum 25(OH)D concentration and LDL cholesterol (*R* = −0.507, *p* < 0.001), Apo-B (*R* = −0.566, *p* < 0.001), Apo-B/Apo-A ratio (*R* = −0.496, *p* < 0.001), total cholesterol (*R* = −0.479, *p* < 0.01), non-HDL cholesterol (*R* = −0.481, *p* < 0.01), and triglycerides (*R* = −0.317, *p* < 0.05) (Table 6). No correlation with HDL cholesterol or Apo-A was found. A decision-tree analysis was created in order to identify which variables might have the greatest prediction power to determine 25(OH)D status. Despite multiple variables being associated with serum 25(OH)D levels in the univariate correlation analysis, after multivariate analysis, Apo-B was the only variable that remained with a significant correlation. An Apo-B level > 76 mg/dL was shown to significantly predict a higher risk of being vitamin D insufficient or deficient (*p* = 0.025) (Figure 4). No seasonal variation was found for any of the lipid parameters in the HFrEF patients, nor was there a correlation between serum 25(OH)D levels and glycemic parameters such as fasting glucose, insulin levels, or HOMA index, or any seasonal variation either.

### 2.5. Inflammatory Cytokines Are Inversely Correlated with 25(OH)D Levels in Patients with Heart Failure

Table 7 shows a strong inverse correlation between serum 25(OH)D levels and the proinflammatory cytokines IL-1β (*R* = −0.779, *p* < 0.001), TNF-α (*R* = −0.530, *p* < 0.001), IL-6 (*R* = −0.418, *p* < 0.01), and IL-8 (*R* = −0.414, p < 0.01), and a weaker negative correlation with IL-17A (*R* = −0.309, *p* < 0.05), IL-18 (*R* = −0.349, *p* < 0.05), and IL-33 (*R* = −0.357, *p* < 0.05) as well. There was no seasonal variation amongst the cytokine milieu. Furthermore, there was no significant correlation between BMI and cytokine levels.

### 2.6. Vitamin D Insufficiency/Deficiency Was Highly Prevalent Throughout All Year

**Figure 5:**
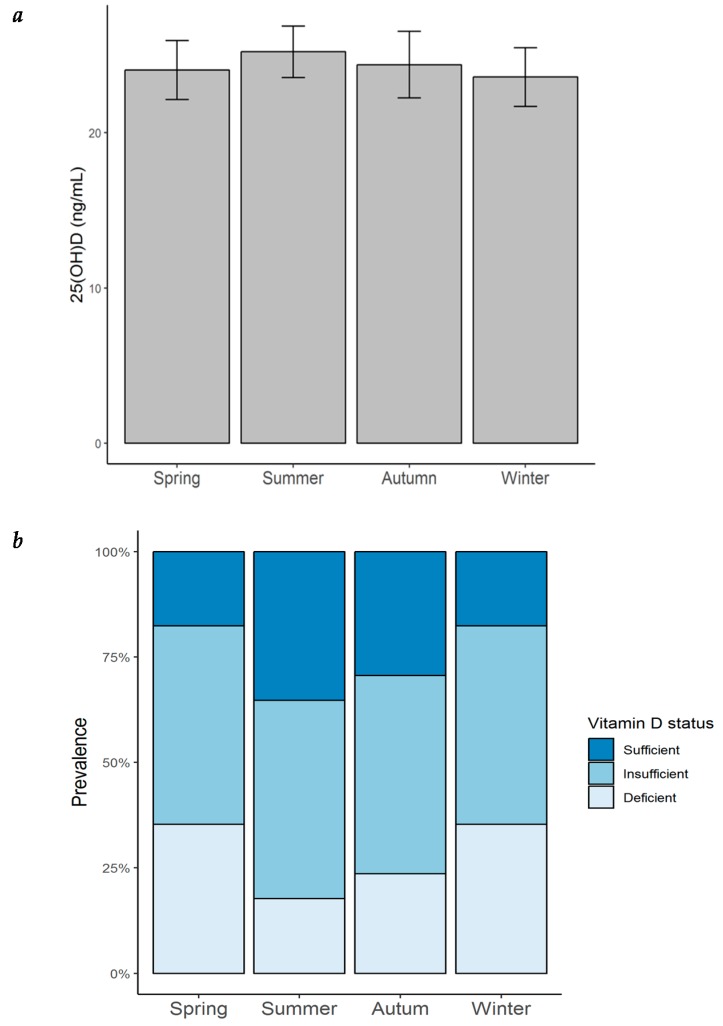
Seasonal variation of serum 25(OH)D concentration and prevalence of vitamin D status in patients with heart failure. (**a**) Seasonal variation of serum 25(OH)D concentration in patients with HF; bar height = mean; error bars = ± standard error; (**b**) prevalence of vitamin D status by season in the same patients. 25(OH)D = 25-hydroxyvitamin D.

Most of the patients with HF in our cohort were vitamin D insufficient or deficient throughout all of the year (Figure 5a). The highest prevalence of vitamin D insufficiency/deficiency was found to be in winter and spring (82.4%), followed by that in autumn (70.6%), and remaining high during the summer (64.7%). An ANOVA analysis was performed to evaluate whether serum 25(OH)D concentrations varied throughout the year. As observed in Figure 5b and Table 8, 25(OH)D levels were found to be lowest in winter (23.6 ± 7.8 ng/mL), followed by spring (24.0 ± 7.8 ng/mL) and autumn (24.4 ± 8.9 ng/mL), while the highest concentrations were found in summer (25.2 ± 6.8 ng/mL). Although no significant seasonal variation was observed, there was a tendency towards a higher prevalence of vitamin D deficiency during winter.

## 3. Discussion

### 3.1. Vitamin D Deficiency in the Setting of Patients with HFrEF

One of the core aspects of recent molecular and clinical research into HFrEF has been to better understand the role of vitamin D and the vitamin D receptor in the initiation and progression of HF. There are several mechanisms underlying this association. Studies in animals and humans have shown that serum 25(OH)D levels are inversely correlated with the RAAS activity, possibly because active vitamin D inhibits renin biosynthesis [25]. Vitamin D deficiency may, therefore, contribute to a hyper-activated RAAS axis, which then promotes the deleterious hemodynamic consequences of salt and water retention, vasoconstriction, and ventricular remodeling. Another mechanism associating vitamin D deficiency and ventricular dysfunction is intracellular calcium handling by the cardiomyocyte. In a mouse model of cardiac dysfunction, mice lacking calcitriol have been found to exhibit abnormal calcium handling, impaired ventricular functioning, and adverse cardiac remodeling and fibrosis [26]. Furthermore, activation of the Vitamin D receptor has been shown to regulate the expression of procollagen 1, which in turn may regulate profibrotic activity in the myocardium [27]. In another animal model, the deletion of the vitamin D receptor gene was also found to activate the calcineurin/nuclear factor of activated T-cells (NFAT) intracellular signaling pathway, a potent pro-hypertrophic signal [28]. Moreover, myocardial contractility may also be enhanced in the presence of sufficient vitamin D levels, which correlates with increased intracellular concentrations of cyclic adenosine monophosphate (cAMP), PKA, and PKC [27]. Overall, these findings demonstrate several mechanistic associations in which vitamin D deficiency may participate in HF progression, hemodynamic abnormalities, and structural dysfunction. Our results show that most of the patients with HFrEF in our cohort have low serum 25(OH)D levels and are in line with other studies. A significant high prevalence of vitamin D deficiency in patients with HF, when comparing low and very low vs. normal serum 25(OH)D levels (HR = 1.33 and 2.19, respectively), has been found [31]. A high prevalence of vitamin D deficiency among patients with HF and an independent association of low 25(OH)D levels with hospitalization and mortality rates have also been described [32]. On the other hand, in a different type of study to evaluate whether inadequate serum levels of 25(OH)D predict the prevalence of chronic conditions such as cardiovascular disease, type 2 diabetes, and obesity, among others, no association with any chronic condition, including heart failure, was found; this study did not report prevalence of vitamin D deficiency [33]. Both cellular and hemodynamic mechanisms may be implicated in this association, which not only explains an increased prevalence of vitamin D deficiency among patients with HF, but may also recognize a connection between vitamin D deficiency and higher risk of adverse outcomes in patients with HF. In our cohort of patients with HF, vitamin D deficiency may thus contribute to ventricular dysfunction, cardiac remodeling, hypertrophy, fibrosis, and disease progression by the molecular and cellular signaling mechanisms described above.

### 3.2. Correlation of Atherosclerotic and Metabolic Parameters with Vitamin D Deficiency in Patients with HFrEF

Diverse types of dyslipidemias have been recognized as risk factors for the development of HF [34], and in conjunction with vitamin D deficiency, they may explain some of the mechanisms for disease progression. Atherosclerosis plays a predominant role in the pathophysiology of ischemic heart disease, which by itself, is the leading cause of HF. LDL-cholesterol particles turn into oxidized LDL cholesterol (oxLDL) by reactive oxygen species. Macrophages, which express high-capacity scavenger receptors that are not down-regulated in the presence of high oxLDL concentration, are modified into foam cells [35]. Clusters of foam cells accumulated in the sub-endothelium, up-regulate the expression of NF-κB, which in turn triggers the membrane expression of vascular cell adhesion protein 1 (VCAM-1) and the endothelial adhesion molecule E-selectin. Another mechanism that increases NF-κB transcription in the endothelial cells is the increased proinflammatory cytokines IL-1, IL-8, and MCP-1 that characterize the inflammatory state in HF [36]. Adhesion molecules VCAM-1 and E-selectin contribute to increased cellular inflammation in the endothelial surface, as macrophages and T-cells are attracted to these proteins [35,36]. This propagates the inflammatory cycle, eventually leading to disturbed blood flow in the vessel’s lumen and progression of atherosclerosis.

**Figure 6:**
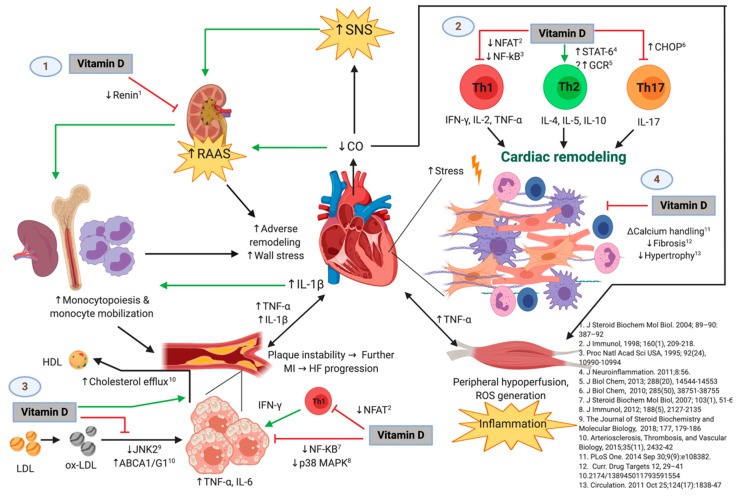
The Possible Immunomodulatory Role of Vitamin D in the Context of Heart Failure (HF) Progression. Green and black lines with arrowheads represent a direct stimulation and a positive correlation, respectively. Red lines ending in perpendicular bars indicate inhibition. (↑) represent an increase in expression, concentration, or activity of the parameter or mechanism. (↓) indicate a decrease in expression, concentration, or activity of the parameter of mechanism. HF is initiated and maintained by chronic maladaptive activation of neurohumoral networks that result from decreased cardiac output (e.g., the sympathetic nervous system [SNS] and the renin-angiotensin-aldosterone system [RAAS]), innate and adaptive immune mechanisms triggered by myocardial damage and stress, and systemic inflammation that results from hypoperfusion of peripheral organs [20,21]. Vitamin D may play a protective role in the progression of HF by interfering with several deleterious pathways [25], which include but are not limited to: (1) inhibiting the release of renin [44], which leads to attenuated RAAS-mediated adverse cardiac remodeling and release of monocytes from bone marrow and lymphoid tissue (an event that is also favored by IL-1β and perhaps other inflammatory cytokines from damaged myocardium) [21]. Monocytes are known to infiltrate damaged myocardium [45] and atheromatous plaques [35], where they exacerbate the local inflammatory response and promote further adverse ventricular remodeling as well as growth and instability of atherosclerotic plaques, respectively. Hence, vitamin D may indirectly attenuate monocyte/macrophage-mediated myocardial damage. (2) Vitamin D may also favor an anti-inflammatory T-helper lymphocyte phenotype by decreasing Th1 [46,47,48] and Th17 [9,23] cytokine production and favoring the Th2 phenotype [49], which would theoretically promote adequate remodeling and decreased fibrosis, and reduce cardiomyocyte hypertrophy and dysfunction. (3) Vitamin D could also slow the progression of atherosclerotic plaques by inhibiting the proinflammatory activation of foam cells (macrophages that have phagocytosed ox-LDL particles) through diminished ox-LDL capture [38], increased cholesterol efflux [37], and decreased activation of transcription factors involved in the expression of proinflammatory cytokines such as TNF-α and IL-6 [22,50]. Like the mechanism described above, vitamin D may also act on T-lymphocytes present in atherosclerotic plaques, decreasing IFN-γ production and hence proinflammatory polarization of macrophages/foam cells. This is of relevance, as growth of atherosclerotic plaques results in plaque instability and further myocardial infarction, with consequent exacerbation of the pathogenic loop that characterizes HF. (4) Vitamin D has also been demonstrated to exert direct effects on the cardiomyocyte and interstitium, including modulation of intracellular calcium handling [26], expression of procollagen-1 [27], and hypertrophic calcineurin/NFAT signaling [28]. Abbreviations: HF = heart failure; MI = myocardial infarction; ABCA1/G1 = ATP-binding cassette transporter types A1 and G1; CHOP = C/EBP homologous protein; CO = cardiac output; GCR = glucocorticoid receptor; IFN = interferon; IL = interleukin; JNK2 = c-Jun N-terminal kinase 2; LDL = low-density lipoprotein; HDL = high-density lipoprotein; NFAT = nuclear factor of activated T-cells; NF-κB = nuclear factor kappa-B; ox-LDL = oxidized low-density lipoprotein; p38 MAPK = p38 mitogen-activated protein kinases; RAAS = renin-angiotensin-aldosterone system; SNS = sympathetic nervous system; STAT6 = signal transducer and activator of transcription 6; Th = T-helper; TNF = tumor necrosis factor-alpha. Original image created with BioRender^®^ (BioRender, Toronto, ON, Canada; website URL: https://biorender.com/; accessed on 9 October 2019).

Regarding these mechanisms, vitamin D may have lipid-regulating effects. Therefore, adequate levels of 25(OH)D might act as a protective factor in coronary disease and HF, but in the case of vitamin D deficiency, there may be deleterious consequences. Figure 6 shows the possible immunomodulatory role of vitamin D regarding inflammation in the context of HF progression. In an animal model of hypercholesterolemia, vitamin D was shown to promote cholesterol efflux from macrophage-derived foam cells by augmenting the expression of ATP-binding membrane cassette transporter types A1 and G1 (ABCA1/G1) [37]. Hepatocyte uptake of cholesterol was also shown to be increased in the presence of higher levels of 25(OH)D concentrations. In addition, vitamin D has also been described to downregulate the c-Jun N-terminal protein kinase 2 (JNK2) cellular pathway in macrophages, which decreases oxLDL uptake and subsequently inhibits transformation into a foam cell phenotype [38]. Given the high prevalence of vitamin D deficiency throughout the four seasons of the year in our cohort of patients with HF, these protective effects of vitamin D may likely be absent. Furthermore, our results show an inverse correlation between 25(OH)D levels and total cholesterol, LDL cholesterol, non-HDL cholesterol, triglycerides, Apo-B, and Apo-B/Apo-A ratio. These correlations may also reflect the mechanisms described above, pointing towards the lack of the possible protective role of vitamin D in the setting of dyslipidemias. Our results are consistent with the prospective study, *The Atherosclerosis Risk in Communities*, in which lower 25(OH)D concentrations were shown to be associated with higher LDL cholesterol and total cholesterol, as well as an overall greater risk of dyslipidemias [39]. Similarly, Forrest et al. found that vitamin D deficiency was significantly more common in individuals with high LDL cholesterol [40].

Dysregulation of glucose metabolism has been linked to vitamin D deficiency and the development, as well as progression, of HF. In patients with established HF, the presence of diabetes mellitus has been associated with increased hospitalization rates and overall increased mortality [34]. Several mechanisms have been described regarding the role of vitamin D in glucose metabolism. In an animal model of vitamin D deficiency, decreased protein kinase B/Akt phosphorylation and reduced expression of glucose transporter 4 (GLUT4) in cardiomyocytes was shown. This, in turn, resulted in insulin resistance by the myocardium, a significant downregulation of the endogenous antioxidant enzymes SOD2 and catalase, and diminished LVEF [41]. The high prevalence of vitamin D deficiency throughout the 12-month follow-up shown in our cohort of patients with HF may thus worsen or predispose them to alterations in glucose metabolism. Moreover, a significant correlation between 25(OH)D levels < 43 ng/mL and impaired fasting glucose (OR = 3.40) and low insulin sensitivity (defined as HOMA index for insulin sensitivity < 90%) (OR = 2.65) has been reported in healthy adults with a median age of 39.4 years old [42]. Specifically, in patients with HF, vitamin D deficiency was shown to be correlated with increased rates of diabetes mellitus when compared with a non-vitamin D deficient group (31.4% vs. 22.8%) [43]. Despite these observations, our results show no correlation between 25(OH)D concentrations and fasting glucose level, insulin concentration, or HOMA index. These findings may be attributed to the low LVEF (below 40%) of all our patients, or to the medications they were taking.

Contrary to what was expected, no correlation was seen between serum 25(OH)D concentrations and LVEF in our cohort. This could be explained by the fact that all of our patients presented abnormally low values of LVEF. Therefore, we could not analyze the whole spectrum of the relationship between vitamin D levels and LVEF.

### 3.3. Inflammation Pathways Associating Vitamin D Deficiency with HFrEF

Figure 6 shows the possible immunomodulatory role of Vitamin D regarding inflammation in the context of HF progression. HFrEF has long been recognized as a systemic proinflammatory state, with pathways involving activation of both innate and adaptive immunity mechanisms [19,20]. Most likely, a crosstalk between the heart and the peripheral organs in which HF promotes inflammation and vice versa might be present [21]. Hemodynamic stress and volume overload have been described to increment wall stress and cell mechanical damage, which stimulates cardiomyocyte release of proinflammatory cytokines, such as TNF-α, IL-1, MCP-1, and IL-6. On the other hand, peripheral organs respond to these inflammatory signals inducing more deleterious effects, such as: skeletal muscle inflammation (which may itself be secondary to chronic vasoconstriction and hypoperfusion due to decreased cardiac output), adipose tissue inflammation, increased atherogenic progression in the endothelium, augmented monocyte production by the bone marrow, and increased bacterial translocation from the gut [21]. This chronic proinflammatory cycle has been shown to contribute to the deterioration of ventricular function by inducing myocardial contractile dysfunction, hypertrophy, apoptosis, and fibrosis, with subsequent progression of HF [51]. Although systemic inflammation is a central pathogenic hallmark of HF [20], it is currently unclear whether vitamin D deficiency plays a specific role in the pathogenesis of HF through the attributed immunomodulatory functions [52]. However, it has been shown that vitamin D deficiency is indeed associated with HFrEF and increased markers of ongoing inflammation [17,53].

In the context of inflammation, the relationship between vitamin D and the cytokine milieu has been shown to represent a core aspect that leads to a proinflammatory state through several mechanisms. Our results in this cohort of patients with HF show serum 25(OH)D levels to be inversely correlated with the proinflammatory cytokines IL-1β, TNF-α, IL-6, and IL-8, although no correlation with the anti-inflammatory cytokine IL-10 was noted. Vitamin D has been found to inhibit the secretion of proinflammatory cytokines TNF-α and IL-6 by monocytes and macrophages through inhibiting p38 MAPK via induction of MKP1, which results in dephosphorylation of p38 [22]. The MAPK pathway is stimulated by diverse stressors to induce expression of proinflammatory cytokines, not only in immune cells but also in the myocardium itself [54]. Since a high prevalence of vitamin D deficiency was found in our study population, induction of this metabolic pathway is highly probable, which might aggravate the inflammatory status that these patients with HFrEF are already in.

Other mechanisms by means of which vitamin D has been associated with inflammation are related to the vitamin D-activated vitamin D receptor/retinoid X receptor (VDR/RXR) heterodimer. VDR/RXR has been reported to interact with transcription factors such as NF-κB, NFAT, and the glucocorticoid receptor (GCR), all of which participate in the transcription of genes involved in inflammatory processes [52]. NF-κB is involved in the transcription of many proinflammatory cytokines, including TNF-α, IL-1β, and IL-6, among others [55], which are direct mediators of cardiac dysfunction [56]. NF-κB expression and activation has been shown to be downregulated by vitamin D [48,50], which may function as a protective mechanism that would not be present in the setting of vitamin D deficiency. In its own way, NFAT upregulates the expression of IL-2, a crucial cytokine for T-cell replication, activation, and induction of Th1-mediated inflammatory responses [47], which appear to be relevant in HF, as evidenced by the higher number of circulating Th1 cells seen in this condition [57]. NFAT signaling has been shown to be inhibited by vitamin D, preventing this transcription factor from binding to its response elements [46], which further indicates another immunological pathway by which vitamin D deficiency may be deleterious in HF. On the other hand, GCR upregulates the expression of anti-inflammatory cytokines, while downregulating proinflammatory ones [58]. Vitamin D has been found to enhance these GCR-mediated anti-inflammatory activities [59], which may represent an anti-inflammatory mechanism that would be absent in circumstances of vitamin D deficiency. Considering the lines of evidence of the mechanisms and pathways provided, low 25(OH)D levels are expected to be accompanied by increased circulating proinflammatory cytokines, as observed in our cohort, which may possibly contribute to clinical deterioration in these patients.

Regarding one of the main pathways of adaptive immunity, our results show serum 25(OH)D concentrations to be negatively correlated with IL-17A as well. Interestingly, vitamin D has also been shown to inhibit the secretion of the proinflammatory cytokine IL-17 by Th17 cells through a post-transcriptional mechanism [23]. Others have shown that vitamin D suppresses autoimmunity, not only by decreasing the production of IL-17, but also by inhibiting the ability of naïve CD4+ T-cells to commit to the Th17 lineage [9]. The anti-Th17 effects of vitamin D could be of pathophysiological relevance in HFrEF, as IL-17 appears to have a role in adverse cardiac remodeling and myocardial fibrosis [60]. Vitamin D seems to induce a shift from a Th1 towards a Th2 phenotype, as well as to suppress Th17 responses [52]. Hence, it is reasonable to assume that vitamin D deficiency exacerbates the deleterious generalized proinflammatory state in our patients with HFrEF. In opposition to a previous study in our group with healthy adults [6], no significant seasonal variation of any cytokine was observed, which is consistent with the permanent state of inflammation that occurs in the context of HF [19,20].

### 3.4. Cluster Analysis

In order to characterize the cytokine profile and the 25(OH)D levels in our patients, we performed a cluster analysis, identifying three clusters. Patients from cluster 1 presented the highest circulating levels of 25(OH)D and the highest dietary intake of vitamin D. They showed the lowest significant levels of the proinflammatory cytokines TNF-α and IL-12p70, and the highest levels of the anti-inflammatory cytokine IL-10, compared with patients from the other two clusters, further reflecting the protective role that vitamin D plays in the cytokine milieu. These patients also showed the lowest concentration of total cholesterol, triglycerides, and Apo-B, representing the possible defensive role of vitamin D in atherosclerosis. Patients from cluster 2 had relatively intermediate levels of vitamin D concentration and vitamin D intake, along with intermediate levels of TNF-α, IL-17A, and IL-10 compared with patients from clusters 1 and 3. Finally, and in opposition, patients from cluster 3 presented the lowest serum 25(OH)D levels. These patients showed the highest concentrations of TNF-α, IL-8, and IL-17A, (significant for TNF-α), and the lowest significant levels of IL-10. They also had the highest levels of total cholesterol, LDL cholesterol, Apo-B, and triglycerides, indicating the proinflammatory and atherosclerotic mechanistic roles of vitamin D deficiency described previously. The role of dietary intake as the main lifestyle factor associated with vitamin D deficiency in this setting of patients may also be considered.

### 3.5. Correlation of Vitamin D Deficiency with Anthropometric and Lifestyle Parameters in Patients with HFrEF

The relationship between vitamin D and anthropometric parameters in the setting of HF has not been thoroughly explored. In the general population, there seems to be a consensus of a negative correlation between serum 25(OH)D concentrations and BMI, WC, and BF% [33,61,62,63,64], mainly attributed to sequestration of vitamin D by adipocytes. However, controversy has been found in patients with HF, as no correlation has been reported in this context between serum 25(OH)D levels and body weight, BMI, mid-arm muscle circumference [65], arm lean mass, leg lean mass, and grip strength (rather related to muscle mass and strength) [66]. Similarly, we found no correlation between 25(OH)D levels and BMI, BF%, and fat mass in kg. On the other hand, in a study with 14 HF patients vs. 14 controls, an inverse relationship was found between 25(OH)D levels and BMI [67]. Our results could be explained by the fact that most of our patients belonged to the overweight BMI and BF% category, and WC was within normal limits. Our sample of patients, like most with HF, are rather not obese because of a higher metabolic waste status due to the disease, and hence do not fully represent each of the BMI or BF% categories.

Likewise, lifestyle factors related to vitamin D status in patients with HF have been understudied. In a questionnaire-based study, patients with HF were less exposed to summer outdoor activity at least once a year compared with controls, but no difference was found in fish consumption (the only food source investigated) or vitamin D supplementation. However, serum 25(OH)D levels were not measured [67]. In the previously mentioned cross-sectional case-control study, that included 14 patients with HF vs. 14 controls, no significant differences in 25(OH)D concentration was reported. HF patients were shown to present less weekly sun exposure, but no differences in daily dietary vitamin D intake between the groups was found [67]. In addition, using data from a large population study, no differences were seen among various amounts of vitamin D intake and the incidence (new cases) of HF, but measurement of 25(OH)D levels was not performed either [68]. Our results during the 12-month follow up showed a significant positive correlation between dietary intake of vitamin D-rich foods and serum 25(OH)D concentrations. Regarding sun exposure, when the total population was considered, no correlation was found between serum 25(OH)D levels and sun exposure. Nevertheless, in a sub-analysis in which patients were separated into two groups, those with any amount of sun exposure versus those with no exposure at all, the prevalence of vitamin D sufficiency was significantly greater in patients exposed to the sun compared with those that had no exposure. Our group previously studied the seasonal variation of serum 25(OH)D concentration in a cohort of 23 healthy subjects with a follow-up of 12 months, in which seasonal variation was observed [6]. However, no seasonal variation of 25(OH)D levels was found in our present study, suggesting that sun exposure may not play a dominant role in vitamin D status (as opposed to healthy controls), but rather vitamin D intake seems to play a greater role. Consideration has to be given to our patients’ skin phototype. Skin pigmentation represents a significant independent risk factor for vitamin D deficiency because UVB is needed in the initial conversion of 7-dehydrocholesterol into cholecalciferol, which is less available for this reaction in darker skin phototypes, as melanin acts as a light-absorbent protein. Thus, our cohort might be prone to vitamin D deficiency, given that the majority of the subjects belonged to phototypes IV and V.

The recommended daily allowance (RDA) of vitamin D for adults 51–70 years old is 600 IU/day, while that of adults >70 years old is 800 IU/day [69]. Therefore, none of the patients in our cohort met the nutritional requirements for vitamin D intake, as all of them had an average intake throughout the year of less than 400 IU/day. Although the time of sun exposure required for vitamin D synthesis varies considerably depending on the living latitude and the season of the year, it has been estimated that 5 to 30 min of sun exposure between 10 a.m. and 3 p.m. at least two times per week may be a reasonable amount [70]. Most of the patients in our cohort (82.3% during summer, autumn, and spring, and 52.9% during winter) obtained at least this level of sun exposure while living at an adequate latitude. Therefore, vitamin D ingestion might be a lifestyle factor as important or even more significant than sun exposure in determining 25(OH)D levels in the setting of our cohort of patients with HF.

The study has some limitations. The sample size is both small and from a Hispanic ethnicity; thus, our results might not be extrapolated to other populations. Hence, results should be cautiously interpreted. Even though data regarding vitamin D-rich foods intake and sun exposure was obtained through a personal interview, the information given by the patients might still be subjective. However, our study has several strengths. This is a 12-month follow up to approach seasonal variation of vitamin D and cytokines. In-vitro and animal model studies concerning the molecular mechanisms were identified to describe and apply these mechanisms to explain our results, given that studies in humans hardly explain them in such detail. We also emphasize these molecular mechanisms in an original figure presented in this article, which could offer insight for further research.

## 4. Materials and Methods

### 4.1. Study Population

We conducted a longitudinal study in a sample of HFrEF patients (49 to 82 years old) following standard treatment. Patients (*n* = 17) were followed during 12 months. Every three months, corresponding to each of the four seasons of the year, every patient was personally interviewed and evaluated for lifestyle, anthropometric, and laboratory parameters. All of the patients live in northeastern Mexico (city of Monterrey, latitude 25°40′0′’N) and are of Hispanic origin. The Fitzpatrick classification of phototypes, the standardized clinical method to evaluate a person’s level of melanin pigmentation, was used to determine the level of skin pigmentation of the patients. Phototype I individuals have fair skin, which burns and peels easily with sun exposure, but does not tan; they usually have light eye and hair color. Phototype VI individuals are dark-skinned; when exposed to the sun, they always tan and never burn [71]. The inclusion criteria were a proved diagnosis of HFrEF (LVEF < 40%) and a New York Heart Association (NYHA) functional class of III or IV [29]. The exclusion criteria were chronic kidney disease, liver disease, LVEF ≥ 40%, chronic use of corticosteroids, ingestion of vitamin D supplements, and being institutionalized. Written informed consent was explained, accepted, and signed by all of the patients. The study was approved on May 14, 2013, by the Ethics and Research Commissions of the School of Medicine of Tecnologico de Monterrey and the Santos y de la Garza Evia Foundation, as well as by the Mexican Secretariat of Health with the project identification code “ESVDIC”.

### 4.2. Vitamin D Intake

Every 3 months, each of the patients were interviewed about regular ingestion of vitamin D-rich foods. Standardized portions of these foods were shown to the patients in order to quantify in a more accurate way the amount of vitamin D ingested per source. The patients were asked about consumption of milk (124 IU/portion), yogurt (120 IU/portion), cheese (20 IU/portion), egg (20 IU/portion), fish (150 IU/portion), and cereal (50 IU/portion). Estimated vitamin D content per food source and portions were determined according to the USDA National Nutrient Database for Standard Reference Release [72].

### 4.3. Sun Exposure

During each visit, patients were asked for the total amount of time being exposed to the sun (minutes per day [mpd] and days per week [dpw]). Activities involving and not involving physical exercise were both taken into account. Skin characteristics according to the Fitzpatrick classification of phototypes were registered [71].

### 4.4. Anthropometric Parameters

For each of the seasons, the following anthropometric parameters were measured: height (m), weight (kg), BMI (kg/m^2^), WC (cm), BF% (%), and fat mass (kg) according to standardized protocols [73]. The body fat percentage was measured by bioimpedance using a TANITA’s BF-350 (Tanita Corporation of America, Inc., Arlington Heights, IL, USA).

### 4.5. Vitamin D, Biochemical Parameters, and Cytokines

The patients had a blood sample withdrawn by venipuncture for each of the seasons. Calcium concentration was obtained by spectrophotometry with the Calcium 3L79-21 kit (06753/R05) (Abbott Laboratories Diagnostics Division, Chicago, IL, USA), while serum 25(OH)D and PTH levels were measured by chemiluminescence with the Vit D25OH 5P02-25 kit (G5-9160/R01) and the iPTH 8K25-25 kit (G6-5257/-R05), respectively, on the Architect iSystem (Abbott Laboratories Diagnostics Division, Chicago, IL, USA). Serum and plasma were also obtained from these samples, centrifuged, and frozen at −80 °C afterward. Vitamin D status was determined according to the Endocrine Clinical Society Guidelines: sufficiency as serum 25(OH)D ≥ 30 ng/mL, insufficiency as serum 25(OH)D ≥ 20 and < 30 ng/mL, and deficiency as serum 25(OH)D < 20 ng/mL [30]. High-sensitivity C-reactive protein levels were measured by quantitative immunoturbidimetric assay with a CRP Vario 6K26-30 and 6K26-41 kits (Abbott Laboratories Diagnostic Division, Chicago, IL, USA). Fasting glucose levels were determined by the hexokinase (HK)/glucose-6-phosphate dehydrogenase (G-6-PD) method with the Glucose 3L82 reagent (DENKA SEIKEN Co. Ltd., Tokyo, Japan). Insulin concentrations were measured by chemiluminescence, using the ARCHITECT Insulin Reagent 8K41-27 kit (Abbot Laboratories Diagnostic Division, IL, USA). Total cholesterol was determined with the Cholesterol 7D62-21 reagent kit (307166/R04) (Abbot Laboratories Diagnostic Division, Chicago, IL, USA) on the Architect cSystem. HDL cholesterol was measured by the accelerator selective detergent method with the Ultra HDL 3K33-21 (307177-R04) assay (Abbot Laboratories Diagnostic Division, Chicago, IL, USA) on the Architect cSystem. Triglycerides were measured via the glycerol-phosphate-oxidase reaction with the Triglyceride 7D74-21 kit (307170/R03) (Abbot Laboratories Diagnostic Division, Chicago, IL, USA) on the Architect cSystem. LDL cholesterol was calculated using the total cholesterol, HDL cholesterol, and triglyceride determinations previously mentioned [74]. Apo-A and Apo-B were obtained via immunoturbidimetric assays with the Apolipoprotein A1 9D92-21 kit (306769/R03) and the Apolipoprotein B 9D93-21 kit (306770/R03), respectively, using the Architect cSystem (Abbot Laboratories Diagnostic Division, Chicago, IL, USA). BNP was measured by chemiluminescence with the BNP 8K28-28 kit (616-010-R01) (Abbott Laboratories Diagnostics Division, Chicago, IL, USA) on the Architect iSystem. Serum was used to obtain a cytokine profile using the Legendplex Human Inflammation Panel through a multianalyte flow cytometry assay (BioLegend, San Diego, CA, USA). This panel allows determination of 13 cytokines: IL-1β, IFN-α, IFN-γ, TNF-α, MCP-1, IL-6, IL-8, IL-10, IL-12p70, IL-17A, IL-18, IL-23, and IL-33. As recommended by Biolegend’s instructions, each experiment was performed in triplicate. The flow cytometer used was FACS-Canto II (Becton Dickinson, Franklin Lakes, NJ, USA). Using the standard curves and the Legendplex Data Analysis software version 7.1 provided by BioLegend (BioLegend, San Diego, CA, USA), the analyte concentration and the serum concentrations were calculated for each of the cytokines.

### 4.6. Subgrouping Analysis by Cluster

In order to better identify which variables may represent a given profile regarding vitamin D deficiency in patients with HFrEF, a cluster analysis by partitioning around medoids (PAM) was performed. A total of 4 observations per patient were included in this analysis (corresponding to each of the seasons). The 25(OH)D levels, inflammatory cytokines, lifestyle factors, biochemical, and anthropometric parameters were included. Differences among clusters were assessed by ANOVA with Tukey’s post-hoc test.

### 4.7. Statistical Analysis

Principal component analysis (PCA) was performed to explore continuous demographic, anthropometric, and lifestyle variables. Seasonal variation was tested with repeated-measures ANOVA. Categorical variables were evaluated with Fisher’s exact test. Univariate association with 25(OH)D concentration was calculated with Spearman’s rank correlation test. A conditional inference tree was constructed for vitamin D status prediction according to parameters with statistically significant association in univariate analysis. The R platform (R package version 2.0.7-1; The R Foundation, Vienna, Austria), along with the cluster [75] and party [76] packages, were used.

## 5. Conclusions

Several lines of evidence establish the mechanistic and metabolic roles of vitamin D deficiency contributing to the inflammatory, immunomodulatory, and atherosclerotic status of patients with HF. Our results in this 12-month follow-up of patients with HFrEF are consistent with previous observations of a high prevalence of vitamin D deficiency in these type of patients. We found no seasonal variation of 25(OH)D levels, nor of the proinflammatory cytokine profile. Serum 25(OH)D concentrations correlated with dietary intake of vitamin D-rich foods. When analyzing our total population, no correlation was seen between 25(OH)D levels and sun exposure; nonetheless, when subgrouping the patients into positive (any amount of sun exposure) and negative (no sun exposure), there was a higher prevalence of vitamin D sufficiency in those belonging to the positive group. Considering the inflammatory state observed in the setting of HF, 25(OH)D levels were inversely correlated with the proinflammatory cytokines IL-1β, TNF-α, IL-6, IL-8, IL-17A, IL-18, and IL-33. Total cholesterol, LDL cholesterol, and triglycerides were also negatively correlated with 25(OH)D concentrations. Cluster analysis to better identify different patient profiles related to vitamin D status revealed that patients with the highest levels of 25(OH)D showed the highest dietary intake of vitamin D-rich foods, along with the lowest concentration of TNF-α, IL-17A, total cholesterol, triglycerides, and Apo-B. Overall, these results point towards the lifestyle factors and lipid parameters related to adequate levels of 25(OH)D. In addition, the protective anti-inflammatory role that vitamin D has in the setting of HFrEF was shown in this group of patients. Some of the pathways underlying these mechanisms have been described mostly in in-vitro and animal models: inhibition of renin biosynthesis; less detrimental ventricular remodeling by inhibition of procollagen-1 expression; increased myocardial contractility; and downregulation of JNK2, calcineurin/NFAT, and NF-κB cellular pathways. Induction of cholesterol efflux from foam cells; increased cholesterol uptake by hepatocytes; adequate regulation of GLUT4 expression in cardiomyocytes; and inhibition of the secretion of proinflammatory cytokines by downregulation of the MAPK pathway, have been attributed to vitamin D. Future studies are necessary in patients with HF to establish the molecular mechanisms involved in the pathogenesis of this disease.

## Figures and Tables

**Table 1:** Serum 25(OH)D levels, demographic, lifestyle, and anthropometric parameters in patients with heart failure.

Parameter	Mean (Standard Deviation)
Age (years)	64.2 (± 8.9)
Gender	Male 82.4%/Female 17.6%
Phototype	III 11.8%/IV 58.8%/V 29.4%
Smoking	Yes 11.8%/No 88.2%
Height (m)	1.6 (± 0.1)
Weight (kg)	76.8 (± 11.7)
Body fat (%)	30.5 (± 6.3)
Fat mass (kg)	23.4 (± 5.6)
Waist circumference (cm)	99.1 (± 9)
BMI (kg/m^2^)	28.5 (± 2.8)
Vitamin D intake (IU/day)	224 (± 113.1)
Sun exposure (min/week)	307.3 (± 444.2)
25(OH)D (ng/mL)	24.3 (± 7.7)

Data are expressed as mean (± standard deviation) unless specified otherwise as a percentage (%). BMI = body mass index; 25(OH)D = 25-hydroxyvitamin D.

**Table 2:** 25(OH)D levels, lifestyle, anthropometrical, and clinical data for patients with heart failure grouped into three clusters according to the PCA analysis.

Parameter	Cluster 1	Cluster 2	Cluster 3
Age (years)	72.2 (± 7.5) ^b, c^	60.4 (± 6.6) ^a^	59.6 (± 6.6) ^a^
Height (m)	1.56 (± 0.06) ^b, c^	1.69 (± 0.06) ^a, c^	1.64 (± 0.06) ^a, b^
Weight (kg)	70.7 (± 9.8) ^b^	85.4 (± 8.0) ^a, c^	66.3 (± 5.4) ^b^
Body fat (%)	35.8 (± 4.9) ^b, c^	29.8 (± 4.3) ^a, c^	22.7 (± 3.3) ^a, b^
Fat mass (kg)	25.4 (± 5.6) ^c^	25.3 (± 2.7) ^c^	15 (± 2.6) ^a, b^
Waist circumference (cm)	98.9 (± 7.7) ^b, c^	103.9 (± 7.1) ^a, c^	87.7 (± 4.2) ^a, b^
BMI (kg/m^2^)	28.9 (± 2.8) ^c^	29.7 (± 1.7) ^c^	24.6 (± 1.2) ^a, b^
Vitamin D intake (IU/day)	322.9 (± 103.4) ^b, c^	173.1 (± 83.9) ^a^	174 (± 72.9) ^a^
Sun exposure (min/week)	202.2 (± 218.9) ^c^	171.7 (± 234.2) ^c^	1178.5 (± 925.6) ^a, b^
Vitamin D (ng/mL)	28.3 (± 8.0) ^b, c^	23.6 (± 7.2) ^a^	19 (± 4.5) ^a^
LVEF (%)	28.8 (± 7.1)	30.5 (± 13.0)	27.3 (± 4.3)

Tukey honest significant difference (HSD); data are expressed as mean (± standard deviation). BMI = body mass index; LVEF = left ventricular ejection fraction. ^a^ = statistical difference when compared vs. cluster 1 (*p* < 0.05); ^b^ = statistical difference when compared vs. cluster 2 (*p* < 0.05); ^c^ = statistical difference when compared vs. cluster 3 (*p* < 0.05). Cluster 1 includes 23 observations from 7 different patients, cluster 2 includes 32 observations from 10 different patients, and cluster 3 includes 13 observations from 4 different patients.

**Table 3:** Biochemical and metabolic parameters in the three clusters of patients with heart failure.

Analyte (Units)	Cluster 1	Cluster 2	Cluster 3
BNP (pg/mL)	125.5 (± 104.1)	138.5 (± 158.9)	183.1 (± 142.3)
hsCRP (mg/dL)	0.5 (± 1.1)	0.5 (± 0.6)	0.1 (± 0.1)
Calcium (mg/dL)	9.4 (± 0.3) ^b, c^	9.2 (± 0.2) ^a^	9 (± 0.3) ^a^
PTH (pg/mL)	80.6 (± 54.0)	61.8 (± 23.4)	58.1 (± 15.2)
Total cholesterol (mg/dL)	138.1 (± 50.2) ^c^	171.5 (± 38.3)	193.9 (± 61.4) ^a^
LDL (mg/dL)	63.5 (± 35.9) ^c^	93 (± 32.5)	127.7 (± 58.2) ^a^
HDL (mg/dL)	38.3 (± 10.8)	42.1 (± 8.5)	41.6 (± 6.8)
non-HDL (mg/dL)	99.8 (± 44.8) ^c^	128.1 (± 31.0)	152.3 (± 58.2) ^a^
VLDL (mg/dL)	30 (± 15.3)	36.4 (± 16.1)	39.8 (± 42.6)
Triglycerides (mg/dL)	149.8 (± 76.7)	181.9 (± 80.7)	199 (± 212.8)
Ratio cholesterol/HDL	3.5 (± 0.6) ^c^	4.1 (± 0.7)	4.7 (± 1.4) ^a^
Apo A (mg/dL)	139.1 (± 16.6)	141.9 (± 20.2)	134.4 (± 11.6)
Apo B (mg/dL)	70.8 (± 26.1) ^b, c^	97.7 (± 18.4) ^a^	101.4 (± 33.4) ^a^
Ratio Apo B/Apo A	0.5 (± 0.2) ^b, c^	0.7 (± 0.1) ^a^	0.7 (± 0.2) ^a^
Glucose (mg/dL)	132.4 (± 50.2)	137.5 (± 54.9)	134 (± 56.4)
Insulin (µU/mL)	16.3 (± 11.3)	20.8 (± 14.7) ^c^	8.6 (± 4.5) ^b^
HOMA index	5.2 (± 4.0)	6.3 (± 3.9) ^c^	2.7 (± 1.6) ^b^

Tukey honest significant difference (HSD); data are expressed as mean (± standard deviation). BNP = brain natriuretic peptide; hsCRP = high sensitivity C-reactive protein; PTH = parathyroid hormone; LDL = low-density lipoprotein; HDL = high-density lipoprotein; non-HDL = non-high-density lipoprotein; VLDL = very low-density lipoprotein; Apo = apolipoprotein; HOMA = Homeostatic Model Assessment. ^a^ = statistical difference when compared vs. cluster 1 (*p* < 0.05); ^b^ = statistical difference when compared vs. cluster 2 (*p* < 0.05); ^c^ = statistical difference when compared vs. cluster 3 (*p* < 0.05).

**Table 4:** Differences in cytokine concentration for the three clusters of patients with heart failure.

Cytokine (pg/mL)	Cluster 1	Cluster 2	Cluster 3
IFN-α2	74.5 (± 99.7) ^b, c^	4.3 (± 6.5) ^a^	1.1 (± 0.4) ^a^
IFN-ɣ	65.7 (± 238.1)	48.6 (± 196.2)	8.2 (± 14.7)
TNF-α	3.3 (± 4.1) ^c^	5.3 (± 5.8)	9.1 (± 3.5) ^a^
MCP-1	543.6 (± 374.9)	490.9 (± 439.4)	252 (± 321.6)
IL-6	6.2 (± 6.9)	3.3 (± 2.1)	4.4 (± 2.6)
IL-8	38.2 (± 82.4)	18.8 (± 34.4)	55.6 (± 117.1)
IL-10	2.7 (± 2.0) ^b, c^	1.1 (± 0.8) ^a^	1 (± 0.9) ^a^
IL-12p70	4.5 (± 6.0) ^b, c^	0.6 (± 0.6) ^a^	0.5 (± 0.4) ^a^
IL-17A	2.4 (± 2.3)	2.8 (± 2.8)	3.5 (± 2.0)
IL-18	471.2 (± 400.6)	284 (± 217.6)	221.9 (± 127.8)
IL-23	36.7 (± 76.8) ^b^	3.6 (± 2.8) ^a^	3 (± 2.2)
IL-33	0.9 (± 0.6)	1 (± 0.9)	2.1 (± 2.7)

Tukey honest significant difference (HSD); data are expressed as mean (± standard deviation). IFN = interferon; TNF = tumor necrosis factor; MCP = monocyte chemoattractant protein; IL = interleukin. ^a^ = statistical difference when compared vs. cluster 1 (*p* < 0.05); ^b^ = statistical difference when compared vs. cluster 2 (*p* < 0.05); ^c^ = statistical difference when compared vs. cluster 3 (*p* < 0.05).

**Table 5:** Association of serum 25(OH)D levels with ventricular function, lifestyle, and anthropometric parameters in patients with heart failure for the 12-month follow-up.

Variable	Spearman’s Rho
Age (years)	0.386 **
Weight (kg)	0.031
Body fat (%)	0.160
Fat mass (kg)	0.150
Waist circumference (cm)	0.150
BMI (kg/m^2^)	0.265 *
Vitamin D intake (IU/day)	0.276 *
Sun exposure (min/week)	−0.075

Spearman’s rank correlation test; * = *p* < 0.05; ** = *p* < 0.01. Values represent the four measurements corresponding to each season of the year per patient (only correlations for serum 25(OH)D levels are shown). LVEF = left ventricular ejection fraction; BMI = body mass index.

**Table 6:** Association of serum 25(OH)D levels with biochemical and metabolic parameters in patients with heart failure for the 12-month follow-up.

Variable	Spearman’s Rho
LVEF (%)	0.251
BNP (pg/mL)	0.221
hsCRP (mg/dL)	0.133
Calcium (mg/dL)	0.354 *
PTH (pg/mL)	0.283
Total cholesterol (mg/dL)	−0.479 **
LDL (mg/dL)	−0.507 ***
HDL (mg/dL)	−0.196
non-HDL (mg/dL)	−0.481 **
VLDL (mg/dL)	−0.317 *
Triglycerides (mg/dL)	−0.317 *
Ratio cholesterol/HDL	−0.423 **
Apo A (mg/dL)	−0.299
Apo B (mg/dL)	−0.566 ***
Ratio Apo B/Apo A	−0.496 ***
Glucose (mg/dL)	−0.073
Insulin (µU/mL)	−0.017
HOMA index	−0.077

Spearman’s rank correlation test; * = *p* < 0.05; ** = *p* < 0.01; *** = *p* < 0.001. Values represent the four measurements corresponding to each season of the year per patient (only correlations for serum 25(OH)D levels are shown). LVEF = left ventricular ejection fraction; BNP = brain natriuretic peptide; hsCRP = high sensitivity C-reactive protein; PTH = parathyroid hormone; LDL = low-density lipoprotein; HDL = high-density lipoprotein; VLDL = very low-density lipoprotein; Apo = apolipoprotein; HOMA = Homeostatic Model Assessment.

**Table 7:** Association of serum 25(OH)D concentration and cytokine levels in patients with heart failure for the 12-month follow-up.

Variable	Spearman’s Rho
IL-1β	−0.779 ***
IFN-α	0.464 ***
IFN-ɣ	−0.046
TNF-α	−0.530 ***
MCP-1	0.257
IL-6	−0.418 **
IL-8	−0.414 **
IL-10	0.001
IL-12p70	0.188
IL-17A	−0.309 *
IL-18	−0.349 *
IL-23	0.114
IL-33	−0.357 *

Spearman’s rank correlation test; * = *p* < 0.05; ** = *p* < 0.01; *** = *p* = 0.001. Values include the four measurements corresponding to each season of the year. IL = interleukin; IFN = interferon; TNF = tumor necrosis factor; MCP = monocyte chemoattractant protein.

**Table 8:** Seasonal variation of serum 25(OH)D levels and prevalence of deficiency, insufficiency, and sufficiency in patients with heart failure.

Season	25(OH)D (ng/mL)	Vitamin D Deficiency *n* (%)	Vitamin D Insufficiency *n* (%)	Vitamin D Sufficiency *n* (%)
Spring	24 (± 7.8)	6 (35.3)	8 (47.1)	3 (17.6)
Summer	25.2 (± 6.8)	3 (17.6)	8 (47.1)	6 (35.3)
Autumn	24.4 (± 8.9)	4 (23.5)	8 (47.1)	5 (29.4)
Winter	23.6 (± 7.8)	6 (35.3)	8 (47.1)	3 (17.6)

Data are expressed as mean (± standard deviation) for serum 25(OH)D levels, and as absolute value (*n*) and percentage (%) of the population for each season. 25(OH)D = 25-hydroxyvitamin D.

## Abbreviations

HF	Heart failure
LDL	Low density lipoprotein
Apo	Apolipoprotein
IL	Interleukin
TNF	Tumor necrosis factor
BMI	Body mass index
UVB	Ultraviolet B-type
25(OH)D	25-hydroxyvitamin D
1,25(OH)2D3	1,25-dihydroxyvitamin D (calcitriol)
PTH	Parathyroid hormone
FGF-23	Fibroblast growth factor 23
WC	Waist circumference
IU	International units
LVEF	Left ventricular ejection fraction
HFrEF	Heart failure with reduced ejection fraction
HFpEF	Heart failure with preserved ejection fraction
HFmrEF	Heart failure with mid-range ejection fraction
RAAS	Renin angiotensin aldosterone system
PK	Protein kinase
NF-κB	Nuclear-factor kappa B
MCP-1	Monocyte chemoattractant protein-1
MAPK	Mitogen-activated protein kinase
MMP	Matrix metalloproteinase
NYHA	New York Heart Association
USDA	United States Department of Agriculture
IFN	Interferon
PCA	Principal component analysis
HSD	Honest significance difference
PMA	Partitioning around medoids
HDL	High density lipoprotein
HOMA	Homeostatic model assessment
BNP	Brain natriuretic peptide
ANOVA	Analysis of variance
cAMP	Cyclic adenosine monophosphate
NFAT	Nuclear factor of activated T-cells
oxLDL	Oxidized low-density lipoprotein
APC	Antigen-presenting cells
JNK2	c-Jun N-terminal protein kinase 2
GLUT4	Glucose transporter 4
VDR/RXR	Vitamin D receptor/Retinoid X receptor
GCR	Glucocorticoid receptor

## References

[1] Cashman K.D. (2019). Vitamin D Deficiency: Defining, Prevalence, Causes, and Strategies of Addressing. Calcif. Tissue Int..

[2] Jeon S.M., Shin E.A. (2018). Exploring vitamin D metabolism and function in cancer. Exp. Mol. Med..

[3] Veldurthy V., Wei R., Campbell M., Lupicki K., Dhawan P., Christakos S. (2016). 25-Hydroxyvitamin D_3_ 24-Hydroxylase: A Key Regulator of 1,25(OH)_2_D_3_ Catabolism and Calcium Homeostasis. Vitam. Horm..

[4] Dhawan P., Peng X., Sutton A.L., MacDonald P.N., Croniger C.M., Trautwein C., Centrella M., McCarthy T.L., Christakos S. (2005). Functional cooperation between CCAAT/enhancer-binding proteins and the vitamin D receptor in regulation of 25-hydroxyvitamin D3 24-hydroxylase. Mol. Cell. Biol..

[5] Holick M.F. (2007). Vitamin D deficiency. N. Engl. J. Med..

[6] Elizondo-Montemayor L., Castillo E.C., Rodríguez-López C., Villarreal-Calderón J.R., Gómez-Carmona M., Tenorio-Martínez S., Nieblas B., García-Rivas G. (2017). Seasonal Variation in Vitamin D in Association with Age, Inflammatory Cytokines, Anthropometric Parameters, and Lifestyle Factors in Older Adults. Mediat. Inflamm..

[7] Herrick K.A., Storandt R.J., Afful J., Pfeiffer C.M., Schleicher R.L., Gahche J.J., Potischman N. Vitamin D status in the United States, 2011–2014. Am. J. Clin. Nutr..

[8] Peterson C.A., Heffernan M.E. (2008). Serum tumor necrosis factor-alpha concentrations are negatively correlated with serum 25(OH)D concentrations in healthy women. J. Inflamm. (Lond.).

[9] Tang J., Zhou R., Luger D., Zhu W., Silver P.B., Grajewski R.S., Su S.B., Chan C.C., Adorini L., Caspi R.R. (2009). Calcitriol suppresses antiretinal autoimmunity through inhibitory effects on the Th17 effector response. J. Immunol..

[10] Rai V., Agrawal D.K. (2017). Role of Vitamin D in Cardiovascular Diseases. Endocrinol. Metab. Clin. N. Am..

[11] Benjamin E.J., Muntner P., Alonso A., Bittencourt M.S., Callaway C.W., Carson A.P., Chamberlain A.M., Chang A.R., Cheng S., Das S.R. (2019). Heart Disease and Stroke Statistics-2019 Update: A Report From the American Heart Association. Circulation.

[12] Savarese G., Lund L.H. (2017). Global Public Health Burden of Heart Failure. Card. Fail. Rev..

[13] Ciapponi A., Alcaraz A., Calderón M., Matta M.G., Chaparro M., Soto N., Bardach A. (2016). Burden of Heart Failure in Latin America: A Systematic Review and Meta-analysis. Rev. Esp. Cardiol. (Engl. Ed.).

[14] Ponikowski P., Voors A.A., Anker S.D., Bueno H., Cleland J.G., Coats A.J., Falk V., González-Juanatey J.R., Harjola V.P., Jankowska E.A. (2016). 2016 ESC Guidelines for the diagnosis and treatment of acute and chronic heart failure: The Task Force for the diagnosis and treatment of acute and chronic heart failure of the European Society of Cardiology (ESC). Developed with the special contribution of the Heart Failure Association (HFA) of the ESC. Eur. J. Heart Fail..

[15] Ziaeian B., Fonarow G.C. (2016). Epidemiology and aetiology of heart failure. Nat. Rev. Cardiol..

[16] Saponaro F., Saba A., Frascarelli S., Frontera C., Clerico A., Scalese M., Sessa M.R., Cetani F., Borsari S., Pardi E. (2018). Vitamin D measurement and effect on outcome in a cohort of patients with heart failure. Endocr. Connect..

[17] D’Amore C., Marsico F., Parente A., Paolillo S., De Martino F., Gargiulo P., Ferrazzano F., De Roberto A.M., La Mura L., Marciano C. (2017). Vitamin D deficiency and clinical outcome in patients with chronic heart failure: A review. Nutr. Metab. Cardiovasc. Dis..

[18] Shirazi L.F., Bissett J., Romeo F., Mehta J.L. (2017). Role of Inflammation in Heart Failure. Curr. Atheroscler. Rep..

[19] Mann D.L. (2015). Innate immunity and the failing heart: The cytokine hypothesis revisited. Circ. Res..

[20] Sánchez-Trujillo L., Vázquez-Garza E., Castillo E.C., García-Rivas G., Torre-Amione G. (2017). Role of Adaptive Immunity in the Development and Progression of Heart Failure: New Evidence. Arch. Med. Res..

[21] Van Linthout S., Tschöpe C. (2017). Inflammation—Cause or Consequence of Heart Failure or Both?. Curr. Heart Fail. Rep..

[22] Zhang Y., Leung D.Y., Richers B.N., Liu Y., Remigio L.K., Riches D.W., Goleva E. (2012). Vitamin D inhibits monocyte/macrophage proinflammatory cytokine production by targeting MAPK phosphatase-1. J. Immunol..

[23] Chang S.H., Chung Y., Dong C. (2010). Vitamin D suppresses Th17 cytokine production by inducing C/EBP homologous protein (CHOP) expression. J. Biol. Chem..

[24] Riek A.E., Oh J., Bernal-Mizrachi C. (2013). 1,25(OH)2 vitamin D suppresses macrophage migration and reverses atherogenic cholesterol metabolism in type 2 diabetic patients. J. Steroid Biochem. Mol. Biol..

[25] Wu M., Xu K., Wu Y., Lin L. (2019). Role of Vitamin D in Patients with Heart Failure with Reduced Ejection Fraction. Am. J. Cardiovasc. Drugs..

[26] Choudhury S., Bae S., Ke Q., Lee J.Y., Singh S.S., St-Arnaud R., Monte F.D., Kang P.M. (2014). Abnormal calcium handling and exaggerated cardiac dysfunction in mice with defective vitamin d signaling. PLoS ONE.

[27] Meems L.M., van der Harst P., van Gilst W.H., de Boer R.A. (2011). Vitamin D biology in heart failure: Molecular mechanisms and systematic review. Curr. Drug Targets.

[28] Chen S., Law C.S., Grigsby C.L., Olsen K., Hong T.T., Zhang Y., Yeghiazarians Y., Gardner D.G. (2011). Cardiomyocyte-specific deletion of the vitamin D receptor gene results in cardiac hypertrophy. Circulation.

[29] Measures J.C.N.Q., The Joint Commission (2016). New York Heart Association (NYHA) Classification.

[30] Holick M.F., Binkley N.C., Bischoff-Ferrari H.A., Gordon C.M., Hanley D.A., Heaney R.P., Murad M.H., Weaver C.M., Society E. (2011). Evaluation, treatment, and prevention of vitamin D deficiency: An Endocrine Society clinical practice guideline. J. Clin. Endocrinol. Metab..

[31] Anderson J.L., May H.T., Horne B.D., Bair T.L., Hall N.L., Carlquist J.F., Lappé D.L., Muhlestein J.B., Group I.H.C.I.S. (2010). Relation of vitamin D deficiency to cardiovascular risk factors, disease status, and incident events in a general healthcare population. Am. J. Cardiol..

[32] Belen E., Sungur A., Sungur M.A. (2016). Vitamin D levels predict hospitalization and mortality in patients with heart failure. Scand. Cardiovasc. J..

[33] Cartier J.L., Kukreja S.C., Barengolts E. (2017). Lower Serum 25-Hydroxyvitamin D Is Associated with Obesity but Not Common Chronic Conditions: An Observational Study of African American and Caucasian Male Veterans. Endocr. Pract..

[34] Bozkurt B., Aguilar D., Deswal A., Dunbar S.B., Francis G.S., Horwich T., Jessup M., Kosiborod M., Pritchett A.M., Ramasubbu K. (2016). Contributory Risk and Management of Comorbidities of Hypertension, Obesity, Diabetes Mellitus, Hyperlipidemia, and Metabolic Syndrome in Chronic Heart Failure: A Scientific Statement From the American Heart Association. Circulation.

[35] Libby P., Buring J.E., Badimon L., Hansson G.K., Deanfield J., Bittencourt M.S., Tokgözoğlu L., Lewis E.F. (2019). Atherosclerosis. Nat. Rev. Dis. Primers..

[36] Gimbrone M.A., García-Cardeña G. (2016). Endothelial Cell Dysfunction and the Pathobiology of Atherosclerosis. Circ. Res..

[37] Yin K., You Y., Swier V., Tang L., Radwan M.M., Pandya A.N., Agrawal D.K. (2015). Vitamin D Protects Against Atherosclerosis via Regulation of Cholesterol Efflux and Macrophage Polarization in Hypercholesterolemic Swine. Arterioscler. Thromb. Vasc. Biol..

[38] Oh J., Riek A.E., Zhang R.M., Williams S.A.S., Darwech I., Bernal-Mizrachi C. (2018). Deletion of JNK2 prevents vitamin-D-deficiency-induced hypertension and atherosclerosis in mice. J. Steroid Biochem. Mol. Biol..

[39] Faridi K.F., Zhao D., Martin S.S., Lupton J.R., Jones S.R., Guallar E., Ballantyne C.M., Lutsey P.L., Michos E.D. (2017). Serum vitamin D and change in lipid levels over 5 y: The Atherosclerosis Risk in Communities study. Nutrition.

[40] Forrest K.Y., Stuhldreher W.L. (2011). Prevalence and correlates of vitamin D deficiency in US adults. Nutr. Res..

[41] Nizami H.L., Katare P., Prabhakar P., Kumar Y., Arava S.K., Chakraborty P., Maulik S.K., Banerjee S.K. (2019). Vitamin D Deficiency in Rats Causes Cardiac Dysfunction by Inducing Myocardial Insulin Resistance. Mol. Nutr. Food Res..

[42] Mayer O., Seidlerová J., Černá V., Kučerová A., Karnosová P., Hronová M., Wohlfahrt P., Fuchsová R., Filipovský J., Cífková R. (2018). Serum Vitamin D Status, Vitamin D Receptor Polymorphism, and Glucose Homeostasis in Healthy Subjects. Horm. Metab. Res..

[43] Cubbon R.M., Lowry J.E., Drozd M., Hall M., Gierula J., Paton M.F., Byrom R., Kearney L.C., Barth J.H., Kearney M.T. (2019). Vitamin D deficiency is an independent predictor of mortality in patients with chronic heart failure. Eur. J. Nutr..

[44] Li Y.C., Qiao G., Uskokovic M., Xiang W., Zheng W., Kong J. (2004). Vitamin D: A negative endocrine regulator of the renin-angiotensin system and blood pressure. J. Steroid Biochem. Mol. Biol..

[45] Epelman S., Liu P.P., Mann D.L. (2015). Role of innate and adaptive immune mechanisms in cardiac injury and repair. Nat. Rev. Immunol..

[46] Takeuchi A., Reddy G.S., Kobayashi T., Okano T., Park J., Sharma S. (1998). Nuclear factor of activated T cells (NFAT) as a molecular target for 1alpha,25-dihydroxyvitamin D3-mediated effects. J. Immunol..

[47] Hermann-Kleiter N., Baier G. (2010). NFAT pulls the strings during CD4± T helper cell effector functions. Blood.

[48] Yu X.P., Bellido T., Manolagas S.C. (1995). Down-regulation of NF-kappa B protein levels in activated human lymphocytes by 1,25-dihydroxyvitamin D3. Proc. Natl. Acad. Sci. USA.

[49] Sloka S., Silva C., Wang J., Yong V.W. (2011). Predominance of Th2 polarization by vitamin D through a STAT6-dependent mechanism. J. Neuroinflamm..

[50] Stio M., Martinesi M., Bruni S., Treves C., Mathieu C., Verstuyf A., d’Albasio G., Bagnoli S., Bonanomi A.G. (2007). The Vitamin D analogue TX 527 blocks NF-kappaB activation in peripheral blood mononuclear cells of patients with Crohn’s disease. J. Steroid Biochem. Mol. Biol..

[51] Zhang Y., Bauersachs J., Langer H.F. (2017). Immune mechanisms in heart failure. Eur. J. Heart Fail..

[52] Wöbke T.K., Sorg B.L., Steinhilber D. (2014). Vitamin D in inflammatory diseases. Front. Physiol..

[53] Liu L.C., Voors A.A., van Veldhuisen D.J., van der Veer E., Belonje A.M., Szymanski M.K., Silljé H.H., van Gilst W.H., Jaarsma T., de Boer R.A. (2011). Vitamin D status and outcomes in heart failure patients. Eur. J. Heart Fail..

[54] Li M., Georgakopoulos D., Lu G., Hester L., Kass D.A., Hasday J., Wang Y. (2005). p38 MAP kinase mediates inflammatory cytokine induction in cardiomyocytes and extracellular matrix remodeling in heart. Circulation.

[55] Liu T., Zhang L., Joo D., Sun S.C. (2017). NF-κB signaling in inflammation. Signal Transduct. Target Ther..

[56] Gullestad L., Ueland T., Vinge L.E., Finsen A., Yndestad A., Aukrust P. (2012). Inflammatory cytokines in heart failure: Mediators and markers. Cardiology.

[57] Fukunaga T., Soejima H., Irie A., Sugamura K., Oe Y., Tanaka T., Kojima S., Sakamoto T., Yoshimura M., Nishimura Y. (2007). Expression of interferon-gamma and interleukin-4 production in CD4± T cells in patients with chronic heart failure. Heart Vessels.

[58] Baschant U., Tuckermann J. (2010). The role of the glucocorticoid receptor in inflammation and immunity. J. Steroid Biochem. Mol. Biol..

[59] Zhang Y., Leung D.Y., Goleva E. (2013). Vitamin D enhances glucocorticoid action in human monocytes: Involvement of granulocyte-macrophage colony-stimulating factor and mediator complex subunit 14. J. Biol. Chem..

[60] Feng W., Li W., Liu W., Wang F., Li Y., Yan W. (2009). IL-17 induces myocardial fibrosis and enhances RANKL/OPG and MMP/TIMP signaling in isoproterenol-induced heart failure. Exp. Mol. Pathol..

[61] Cheng S., Massaro J.M., Fox C.S., Larson M.G., Keyes M.J., McCabe E.L., Robins S.J., O’Donnell C.J., Hoffmann U., Jacques P.F. (2010). Adiposity, cardiometabolic risk, and vitamin D status: The Framingham Heart Study. Diabetes.

[62] Pantovic A., Zec M., Zekovic M., Obrenovic R., Stankovic S., Glibetic M. (2019). Vitamin D Is Inversely Related to Obesity: Cross-Sectional Study in a Small Cohort of Serbian Adults. J. Am. Coll. Nutr..

[63] Gu J.K., Charles L.E., Millen A.E., Violanti J.M., Ma C.C., Jenkins E., Andrew M.E. (2019). Associations between adiposity measures and 25-hydroxyvitamin D among police officers. Am. J. Hum Biol..

[64] Hannemann A., Thuesen B.H., Friedrich N., Völzke H., Steveling A., Ittermann T., Hegenscheid K., Nauck M., Linneberg A., Wallaschofski H. (2015). Adiposity measures and vitamin D concentrations in Northeast Germany and Denmark. Nutr. Metab. (Lond.).

[65] Price R.J., Witham M.D., McMurdo M.E. (2007). Defining the nutritional status and dietary intake of older heart failure patients. Eur. J. Cardiovasc. Nurs..

[66] Loncar G., Bozic B., von Haehling S., Düngen H.D., Prodanovic N., Lainscak M., Arandjelovic A., Dimkovic S., Radojicic Z., Popovic V. (2013). Association of adiponectin with peripheral muscle status in elderly patients with heart failure. Eur. J. Intern. Med..

[67] DiCarlo C., Schmotzer B., Vest M., Boxer R. (2012). Body mass index and 25 hydroxyvitamin D status in patients with and without heart failure. Congest. Heart Fail..

[68] Robbins J., Petrone A.B., Gaziano J.M., Djoussé L. (2016). Dietary vitamin D and risk of heart failure in the Physicians’ Health Study. Clin. Nutr..

[69] National Academy of Sciences (2011). Dietary Reference Intakes for Adequacy: Calcium and Vitamin D.

[70] National Institutes of Health, ODS (2019). Vitamin D: Fact Sheet for Health Professionals.

[71] U.S. Department of Health & Human Services (2015). USDA National Nutrient Database for Standard Reference Release 28.

[72] Fitzpatrick T.B. (1988). The validity and practicality of sun-reactive skin types I through VI. Arch. Dermatol..

[73] Centers for Disease Control and Prevention (2017). National Health and Nutrition Examination Survey (NHANES): Anthropometry Procedures Manual.

[74] Krishnaveni P., Gowda V.M.N. (2015). Assessing the Validity of Friedewald’s Formula and Anandraja’s Formula For Serum LDL-Cholesterol Calculation. J. Clin. Diagn. Res..

[75] Maechler M., Rousseeuw P., Struyf A., Hubert M., Hornik K. (2018). Cluster: Cluster Analysis Basics and Extensions.

[76] Hothorn T., Hornik K., Zeileis A. (2006). Unbiased Recursive Partitioning: A Conditional Inference Framework. J. Comput. Graph. Stat..

